# Hepatitis B virus infection: seroprevalence, knowledge, attitude and practice among healthcare workers at Abu-Salim trauma hospital, Tripoli-Libya

**DOI:** 10.11604/pamj.2026.53.10.49883

**Published:** 2026-01-12

**Authors:** Osamah Mohamed Alghizeewi, Elloulu Taher BenDarif, Tarek Mohamed Gibreel, Haytham Mahmoud Al-Salimi, Amal Ben Drief

**Affiliations:** 1 Department of Surgery, Abu-Salim Trauma Hospital, Tripoli, Libya,; 2 Department of Medical Microbiology and Immunology, Faculty of Medicine, University of Tripoli, Tripoli, Libya,; 3 Department of Medicine, Faculty of Medicine, University of Tripoli, Tripoli, Libya,; 4 Department of Medicine, Maitiga Hospital, Tripoli, Libya

**Keywords:** Hepatitis B virus, healthcare, knowledge, attitude, practice

## Abstract

**Introduction:**

healthcare workers (HCWs) are at an increased risk of occupational acquisition of Hepatitis B Virus (HBV) infection. Therefore, this study aimed to determine the seroprevalence and awareness of HBV infection among the HCWs in Abu-Salim Trauma Hospital, Tripoli, Libya.

**Methods:**

a cross-sectional study was carried out on 206 HCWs at Abu-Salim Trauma Hospital from February to March 2022. Serum samples were tested to detect HBsAg, HBcAb, HBeAb and HBsAb. A structured questionnaire was used to assess knowledge, attitudes, and practices (KAP) and a cut-off of 75% was established to categorize KAP scores. Pearson´s chi-square test was used to evaluate the relationship between HBV marker rates and categorical variables, while KAP scores were assessed using the Kruskal-Wallis test.

**Results:**

the prevalence of HBsAg, HBsAb, HBcAb and HBeAb among 206 HCWs was 0%, 50.4%, 3.9%, and 2.9%, respectively. High rates of HBcAb and HBsAb were significantly associated with more than 5 years of professional experience (P= 0.002 and P= 0.0008, respectively). 50.4% of participants were immunized by previous vaccinations. The rates of scores for knowledge, attitude, and practice regarding HBV infection were 62.5%, 72.8%, and 84%, respectively. The mean scores for participants´ knowledge and attitudes concerning HBV infection were significantly associated with their gender, age groups, occupation, and educational level (P= 0.023, 0.045, 0.001, and 0.002) and (P= 0.002, 0.000, 0.000, and 0.000), respectively.

**Conclusion:**

the study showed a low prevalence of past HBV infection and about half of participants were unvaccinated. The mean scores for KAP assessment were reflected insufficient knowledge, weakly positive attitude and good adherence to practices.

## Introduction

The hepatitis B virus (HBV) is a bloodborne pathogen that has emerged as a significant global public health challenge. The majority of individuals infected with HBV may encounter serious complications including liver cirrhosis, hepatocellular carcinoma, liver failure and ultimately death [1,2]. World Health Organization (WHO) estimated 254 million people are living with HBV, with the highest prevalence observed in the African regions, accounting for 25.4% of cases [3]. Healthcare workers (HCWs) are particularly exposed to HBV infection during their professional activities, facing a risk that is up to four times greater than general population. Among the 35 million HCWs worldwide, approximately 3 million are exposed to HBV each year, leading to an estimated 66,000 new infections and 261 fatalities [4,5]. This situation facilitates the ongoing transmission of HBV between patients and HCWs, as well as to HCWs relatives [6]. Therefore, HCWs play a vital role in preventing and controlling HBV infection by increasing the awareness among the public about HBV and promoting behavior changes that can help prevent infectious diseases [7]. The global prevalence of HBsAg, HBsAb, HBcAb and HBeAg among HCWs was 2.3%, 56.6%, 9.2% and 0.2%, respectively [8]. Two national studies have shown that the prevalence of HBsAg among HCWs in the eastern region of Libya is 4.9% [9], while in the western region it was significantly lower (1.1%) [10]. Additional studies conducted by Elzouki *et al*. and Ziglam *et al*. in the western and eastern regions of Libya found that the prevalence rates of HBcAb among HCWs were 8.5% and 17.3%, respectively [10,11].

The prevalence of HBV infection can serve as an indicator for various risk factors associated with HBV transmission, including occupational injuries and lack of vaccination [12]. Elzouki *et al*. reported a high rate of needle stick injuries and a low hepatitis B vaccination rate among HCWs in three referral hospitals in Libya [13]. However, several studies examined HBV vaccination coverage among HCWs in Africa, reported that full coverage ranged between 0.8% in Rwanda to 60.2% in Libya [10,14]. The high vaccination coverage among Libyan population can be attributed to the mandatory vaccination program against HBV infection for newborns and adolescents, which was introduced in 1989 [15]. In addition, to reduce the risk of infection for both HCWs and the community, HCWs are required to maintain a high level of awareness regarding HBV [16]. HCWs in developing countries exhibit limited awareness related to HBV infection. This gap in knowledge directly contributed to inadequate preventive measures, including lower rates of HBV vaccination among HCWs [17]. Several studies highlighted these deficiencies across various African countries, with KAP rates estimated at 25% in Tanzania, 73.9% in Ethiopia, 43.3% in Nigeria and 67.9% in Libya [11,18,19]. In the western region of Libya, studies on the prevalence of HBV infection and the awareness levels among HCWs remain limited. Therefore, this study was designed to investigate the epidemiology of HBV by assessing the seroprevalence of HBV infection and level of knowledge, attitude and practice among HCWs toward HBV infection in the Abu-Salim Trauma Hospital, Tripoli-Libya.

## Methods

**Study design and setting:** a cross-sectional study was designed as a seroepidemiological survey among HCWs from February to March 2024 at Abu-Salim Trauma Hospital in Tripoli, Libya. The hospital has a capacity of 480 beds and employs 645 HCWs. The study was conducted across various hospital departments, including surgical units, the emergency room (ER), the intensive care unit (ICU), and a laboratory.

**Participants:** a total of 206 HCWs (32%) of hospital HCWs were recruited for the study, including 86 (41.7%) physicians, 83 (40.3%) nurses, and 37 (17.9%) technicians. Inclusion criteria for the study included HCWs who had been employed at the current health facility for at least one year at the time of data collection, were 18 years of age or older, had contact with blood and body fluids, handled sharp materials, and belonged to one of three professional categories (Nurses, doctors or laboratory staff).

**Study size:** the study size was calculated using Fisher's formula [20]:


$$N=\frac{Z^{2}P\left(1-P\right)}{I^{2}}$$

To determine the minimum sample size (N) for the study, z is the z-score corresponding to a 95% confidence interval (1.96), P is the expected prevalence of the HBsAg among HCWs in the western region of Libya (1.1%) [10], and l is the margin of error set at 5%.

**Detection of serological markers:** serum samples from all participants were analyzed for HBsAg using automated immunoassay technique (UniCel DxI 600 Access Immunoassay System, Beckman Coulter, USA), whereas, HBeAb, HBsAb and HBcAb markers were detected (using Snibe Maglumi 2000 Chemiluminescence Immunoassay (CLIA) System, China).

**Data collection:** a questionnaire form was designed following Karunamoorthi's guidelines to gather demographic information, including age, gender, education level, occupation, and years of experience [20]. In addition, it covered HCWs' knowledge about HBV etiology, symptoms, transmission routes, disease outcomes, treatment and prevention. The questionnaire also explored HCWs' attitudes toward preventing HBV transmission and their vaccination status. Furthermore, it evaluated HCWs' practices related to infection prevention and control measures. The questionnaire consists of 24 questions: 12 for knowledge, 7 for attitude, and 5 for practice. Each correct answer awarded 1 point, while an incorrect answer received 0 points. The total number of correct answers from each participant was used to establish thresholds for each section of the KAP. A modified Bloom´s cutoff value was used to categorize participants´ knowledge, attitudes and practice [21-24]. Obtaining at least 75% of the maximum score was considered to be a good score.

**Statistical analysis:** Fisher's Exact Test was used to evaluate the association of demographic variables with seroprevalence. P-values ≤ 0.05 were considered statistically significant. For KAP data, Kruskal Wallis test was used to derive statistical inferences (P < 0.05).

**Ethical approval:** it was granted by Libyan Academy of Graduate Studies, Tripoli and National Center for Disease Control (NCDC), Tripoli with protocol number (Ref N®: NBC: 002. H-24.8). Written informed consent was taken from all participants.

## Results

**Participants:** a total of 206 HCWs participated in the survey, with ages ranging from 18 to 58 years (mean age: 34.3 ± SD 7.7 years). The participants were divided into three age groups (≤30, 31-40 and > 40 years) (Table 1). The majority of participants were between 31 and 40 years old (41.7%). Furthermore, most of the participants were females (63.1%), resulting in an overall gender ratio of 1.7: 1. The distribution of participants by occupation reveals that physicians and nurses are nearly equal in representation, 41.7% and 40.3%, respectively, whereas the technicians represent 17.9% of the participants (Table 1).

**Table 1:** prevalence of HBV markers among various demographic data

Variables		Investigated No. (%)	Positive HBcAb No. (%)	P-value	Positive HBsAb No. (%)	p-value	Positive HBeAb No. (%)	P-value
Gender	Female	130 (63.1)	5 (3.8)	1.000	65 (50)	0.1	4 (3.0)	1.000
	Male	76 (36.9)	3 (3.9)		47 (61.8)		2 (2.6)	
Age group	≤ 30	75 (36.4)	0	0.05*	30 (40)	0.002*	0	0.028*
	31- 40	86 (41.7)	3 (3.4)	1.0000	52 (60.4)	0.15	2 (2.3)	1.000
	> 40	45 (21.8)	5 (11.1)	0.01*	30 (66.6)	0.06	4 (8.8)	0.021*
Marital status	Single	116 (56.3)	3 (2.5)	0.3	59 (50.8)	0.26	3 (2.5)	1.000
	Married	90 (43.6)	5 (5.5)		53 (58.8)		3 (3.3)	
Years of experience	≤ 5 years	105 (50.9)	0	0.002*	45 (42.8)	0.0008*	0	0.012*
	> 5 years	101 (49.1)	8 (7.9)		67 (66.3)		6 (5.9)	
Occupation	Physician	86 (41.7)	3 (3.4)	1.000	56 (65.1)	0.01*	2 (2.3)	1.000
	Nurse	83 (40.3)	4 (4.8)	0.30	41 (49.3)	0.41	3 (3.6)	0.68
	Technician	37 (18)	1 (2.7)	0.15	15 (40.5)	1.10	1 (2.7)	1.000
Level of education	Bachelors	123 (59.7)	4 (3.2)	0.71	71 (57.7)	0.25	3 (2.4)	0.68
	High diploma	83 (40.3)	4 (4.8)		41 (49.3)		3 (3.6)	

* Fisher's exact test (P < 0.05)

**Prevalence of hepatitis B virus markers among healthcare workers:** the participants were screened for various hepatitis B markers, including HBsAg, HBcAb, HBsAb, and HBeAb. The presence of any of these markers indicated a state of infection, recovery, or immunization as shown in Table 2. The overall seroprevalence of past HBV infection was 3.9% of the participants. Two participants (0.9%) were immune due to previous natural exposure, while 6 participants (2.9%) were identified as convalescent from HBV infection. Additionally, 104 participants (50.4%) had received immunization from previous vaccinations (Table 2).

**Table 2:** patterns of serological markers of HBV among the 206 HCWs in Abu-Salim Hospital

Serological marker					Interpretation	No. of positive (%)
HBsAg	HBsAb	HBcAb	HBeAb	HCV-Ab		
Negative	Negative	Negative	Negative	Negative	Susceptible	94 (45.6)
Negative	Positive	Positive	Positive	Negative	Convalescent of HBV infection	6 (2.9)
Negative	Positive	Positive	Negative	Negative	Immune due to previous natural exposure	2 (0.9)
Negative	Positive	Negative	Negative	Negative	Immunized by vaccination	104 (50.4)

**Socio-demographic data associated with hepatitis B virus markers:** the prevalence of positive HBcAb, HBsAb and HBeAb markers among different demographic variables is shown in Table 1. There was a significant association between high rates of HBcAb and HBeAb with age group > 40 (P= 0.01 and 0.021), respectively. On the other hand, significant association between high rates of HBcAb, HBsAb and HBeAb with more than 5 years of experience (P= 0.002, 0.0008 and 0.012), respectively were also detected. Furthermore, a significant association was detected between high rate of HBsAb and physician (P= 0.01) (Table 1).

**Assessment of immunization status among healthcare workers:** the number of participants who reported receiving HBV vaccine was 94 (45.6%), compared to 112 participants (54.3%) who reported not receiving HBV vaccine. Based on participants´ reported vaccination status, 60 (63.8%) of the vaccinated HCWs and 52 (46.4%) of reported unvaccinated participants had detectable HBsAb. Moreover, 34 participants (36.1%) who reported being vaccinated did not have detectable HBsAb.

**Assessment of knowledge, attitude and practice toward hepatitis B virus:** a twelve-question survey was conducted to assess HCWs' knowledge of HBV infection. Among the 206 HCWs surveyed, 144 (70.3%) demonstrated good knowledge regarding the transmission, prevention, treatment, and prognosis of HBV infections. 80% of physicians exhibited good knowledge, while technicians and nurses with good knowledge were 69% and 62%, respectively. Most HCWs correctly identified that HBV can be transmitted through blood and needle-stick injuries, with response rates of 88.3% and 87.3%, respectively. However, the lowest correct response rate was noted for the question regarding vertical transmission of HBV, which was only 54.8%. While most HCWs expressed a positive attitude towards HBV infection, only 45.6% had received the HBV vaccine. Among them, nurses had the lowest vaccination rate (27.7%). Additionally, 65% of HCWs reported being screened for HBV, but only 50.6% of the nursing team was screened. The overall positive attitude response rate among HCWs was 73.7%, with nurses showing the lowest rate (65.2%). The survey also revealed that 85.7% of respondents practiced safe hygiene methods concerning HBV infection. The lowest rate of reported needle-stick injuries was among medical physicians (52.3%), while the overall positive response rate regarding safety practices among HCWs was (66.9%).

**Association between demographic characteristics with knowledge, attitudes, and practices scores:** the participants achieved an overall mean score for HBV knowledge 7.5 (62.5%), with statistically significant differences noted across categories such as gender, age group, occupation, level of education, and departments (P= 0.023, 0.045, 0.001, 0.002, and 0.043), respectively. The mean positive attitude score for all participants was 5.1 (72.8%), with significant variations observed among gender, age group, years of experience, occupation, and education level, resulting in P-values of 0.002, 0.000, 0.005, 0.000, and 0.000, respectively. The participants' mean of good practice score was 4.2 (84%), with significant difference observed only in relation to gender (P= 0.005), as shown in Table 3.

**Table 3:** knowledge, attitudes, and practices mean scores of HCWs among various demographic characteristics among Ab-Salim Trauma Hospital HCWs in Tripoli-Libya

Variables		Investigated No. (%)	Knowledge score (±SD)	p-value	Attitude score (±SD)	p-value	Practice score (± SD)	p-value
All participants		206	7.5 (2.9)	-	5.1 (1.8)	-	4.2 (1.0)	-
Gender	Female	130 (63.1)	7.06 (3.2)	0.023*	4.87 (1.9)	0.002*	4.38 (0.9)	0.005*
	Male	76 (36.9)	8.25 (2.1)		5.65 (1.6)		4.01 (1.1)	
Age groups	≤ 30	75 (36.4)	6.82 (3.2)	0.045*	4.56 (1.9)	0.000*	4.06 (1.2)	0.07
	31- 40	86 (41.7)	8.03 (2.2)		5.65 (1.7)		4.30 (0.8)	
	> 40	45 (21.8)	7.60 (3.2)		5.24 (1.7)		4.44 (1.0)	
Marital status	Single	116 (56.3)	7.61 (3.0)	0.37	5.04 (2.0)	0.52	4.17 (1.1)	0.37
	Married	90 (43.7)	7.35 (2.7)		5.32 (1.6)		4.34 (0.9)	
Years of experience	≤ 5 years	105 (50.9)	7.31 (3.0)	0.28	4.85 (1.9)	0.005*	4.11 (1.1)	0.061
	> 5 years	101 (49.1)	7.69 (2.7)		5.48 (1.7)		4.38 (0.9)	
Occupation	Physician	86 (41.7)	8.51 (1.8)	0.001*	5.89 (1.4)	0.000*	4.19 (0.9)	0.223
	Nurse	83 (40.3)	6.46 (3.7)		4.38 (2.0)		4.30 (1.1)	
	Technician	37 (18)	7.45 (2.1)		5.21 (1.6)		4.24 (1.1)	
Level of education	Bachelors	123 (59.7)	8.19 (1.9)	0.002*	5.69 (1.5)	0.000*	4.21 (1.0)	0.168
	High diploma	83 (40.3)	6.46 (3.7)		4.38 (2.0)		4.30 (1.1)	

* Kruskal Wallis test (P < 0.05)

## Discussion

This study evaluated the prevalence of HBV markers and assessed the knowledge, attitude and practice of HCWs in an emergency health facility in Tripoli, Libya. The findings provide critical insight into HCWs exposure risks and potential gaps in knowledge, attitude and practice among HCWs. This study found that none of the HCWs tested positive for HBsAg. This finding contrasts with local studies carried out by Elzouki *et al*. who reported the prevalence of chronic HBV infection were 1.8% and 4.9% among HCWs in the eastern region of Libya, respectively [11,13]. Another national survey conducted in western region of Libya by Ziglam *et al*. among HCWs in Tripoli Central Hospital reported a prevalence of chronic HBV infection (1.1%) [10]. The prevalence of past HBV infection in HCWs participants was 3.9%. Two previous studies carried out by Elzouki *et al*. and Ziglam *et al*. reported that the prevalence rates of HBcAb were 8.5% and 17.3% among HCWs, respectively [10,11]. This variation in the prevalence of chronic and past HBV infection among HCWs in different hospitals in Libya could be attributed to recent enforcement of infection prevention practice in healthcare facilities. The seroprevalence of total immunity acquired by natural HBV infection was 0.2%, which is considerably lower than the global estimate of 9.2% reported among HCWs by Mahamat *et al*. [25]. This marked difference may be attributed to variations in HBV endemicity, occupational exposure risks. The prevalence of HBeAb was 2.9% compared to Elzouki *et al*. who reported a prevalence of 8.0% among HCWs in five major hospitals in eastern region of Libya [11]. The low HBeAb rate could be attributed to reduce in HBV infection rate, possibly due to effective vaccination strategies and strengthened infection prevention and control measures.

HBsAb is considered a marker of immunity against HBV infection, acquired through either HBV vaccination or successful recovery from a prior infection. The study found that 50.4% of the participants were successfully vaccinated, closely aligning with the 51.4% reported by Elzouki *et al*. and considerably higher than the 21.8% documented by Ziglam *et al*. [10,11]. However, these indicate that nearly half of HCWs either remain unvaccinated or have incomplete vaccination records. There is a need for mandatory vaccination of HCWs instead of leaving the responsibility for vaccination schedules to the employees. The association of HBcAb rate across different age groups showed a significantly higher rate among participants over 40 years old compared to the other groups (11.1%, P= 0.01). The higher prevalence may be attributed to lack of an HBV vaccination program for this age cohort in Libyan society. Meanwhile, the distribution of HBsAb across age groups demonstrated a significantly lower prevalence among participants aged 30 years and below (40%, P= 0.002). The reduced rate of HBs in this younger age group may be attributable to incomplete vaccination schedules or suboptimal adherence to the HBV vaccine series. In relation to work experience, the HCWs with more than five years of experience had significantly higher prevalence rates of HBcAb (7.9%, P= 0.02) and HBsAb (66.3%, P= 0.0008). These results are generally consistent with (11.5% and 57.9%) respectively, reported by Elzouki *et al*. among HCWs with similar years of experience in hospitals in eastern region of Libya [11]. The slight differences between the two studies may be due to variations in vaccination coverage, occupational exposure, or infection control measures in different regions.

In a comparison of occupational categories, physicians demonstrated a significantly higher prevalence of HBsAb (65.1%, P= 0.01) compared to paramedic teams. Among the physicians, 62.7% (54/86) reported receiving HBV vaccine. Of these, 63% exhibited immunity attributable to vaccination, while 5.5% had immunity resulting from natural HBV exposure. In contrast, the majority of nurses (72.2%, 60/83) reported being unvaccinated. Of those reported being unvaccinated, 45% were immune through vaccination, while 5% had immunity due to natural exposure to HBV. This finding suggests that a considerable proportion of nurses may have been unaware of their vaccination status, possibly because they had received HBV immunization as part of the routine childhood vaccination program. Overall, HBsAb positivity was observed in 63% of participants who reported being vaccinated and in 46.4% of those who reported being unvaccinated. These findings contrast with the results of Elzouki *et al*. in the eastern region of Libya, where 98% of HCWs who reported receiving the HBV vaccine tested positive for HBsAb [11]. Overall, the mean KAP score of the participants was 16.8 out of 24, representing 70% of the maximum possible score. However, the mean scores for knowledge, attitude and practice were 7.5 out of 12 (62.5% of the maximum score), 5.1 out of 7 (72.8% of the maximum score), and 4.2 out of 5 (84% of the maximum score), respectively. The scores showed that the participants had low knowledge and attitude but demonstrated good practice. These findings align with a previous survey by Elfaitouri *et al*. conducted at the Benghazi Medical Center, which reported maximum scores for knowledge, attitude and practice of 67.2%, 70.7% and 87.5%, respectively [26].

The mean scores for knowledge, attitude and practice were 8.25, 5.65 and 4.01 for males and were 7.06, 4.87 and 4.37 for females, respectively. These differences were statistically significant (P= 0.023, 0.002 and 0.005), respectively. These findings are consistent with those of Yakudima *et al*. and Abalkhail *et al*. who observed that males exhibited greater knowledge and practice than females [27,28]. This may be due to the tendency of males to be more involved in discussions about these topics than females. The significant association of age groups with mean scores for knowledge and attitude (P= 0.045 and 0.000), respectively, could be explained by high knowledge score (8.03) and attitude score (5.65) among age group participants (31-40). This aligns with a survey conducted in Liberia by Freeman *et al*. which found a significant correlation between >35 years participants and high knowledge scores regarding HBV infection (P= 0.0045) [29]. Based on years of experience, the analysis showed a statistical difference (P= 0.005) in mean attitude scores. The present data indicate that participants with over 5 years of professional experience demonstrated a higher mean attitude score (5.48) compared to those with less experience. This outcome is consistent with the findings of Abalkhail *et al*. who reported that HCWs with more than 6 years of experience exhibited both greater knowledge and more favorable attitudes regarding HBV infection compared to their less-experienced counterparts [28]. The correlation between participants' knowledge and attitudes with their occupation and educational levels indicated that physicians and bachelor degree holders achieved significantly higher mean scores in both knowledge and attitudes than nurses and technicians (P= 0.001 and 0.002) and (P= 0.000 and 0.000), respectively. This result is similar to a study conducted among HCWs in White Nile State, Sudan, which found a significant association of knowledge level with both occupation and educational degree [30]. The high score of knowledge and attitudes among physicians may be attributed to the more comprehensive health education programs concerning viral hepatitis that are integrated into medical training compared to those in other educational frameworks.

## Conclusion

The study showed zero prevalence of chronic hepatitis B infection and low prevalence rate of past hepatitis B infection; this suggests either a low endemicity of HBV or efficacy of institutional infection control practices at Abu-Salim Trauma Hospital. Approximately half of the HCWs were either unvaccinated or had incomplete vaccination. This highlights the need for enforcing mandatory vaccination programs. Additionally, the study emphasizes the necessity for targeted educational interventions to enhance knowledge and attitudes about the virus, particularly among nurses and less-educated staff.

### What is known about this topic

- Healthcare workers are at a significantly increased risk for HBV infection due to occupational exposure;
- Vaccination remains the most effective preventive measure against the HBV among HCWs;
- Educational interventions have proven effective in improving awareness and understanding of HBV transmission.

### What this study adds

- The study reports the seroprevalence of HBV infection among HCWs at Abu-Salim Trauma Hospital in Tripoli, Libya;
- It highlights a critical gap in preventive coverage, showing that nearly half of HCWs are either unvaccinated or have incomplete records;
- Despite low scores in knowledge and attitude regarding HBV, many HCWs reported high scores of preventive practice, indicating disconnect that warrants further investigation.
